# Acceleration of chemical shift encoding-based water fat MRI for liver proton density fat fraction and T2* mapping using compressed sensing

**DOI:** 10.1371/journal.pone.0224988

**Published:** 2019-11-15

**Authors:** Fabian K. Lohöfer, Georgios A. Kaissis, Christina Müller-Leisse, Daniela Franz, Christoph Katemann, Andreas Hock, Johannes M. Peeters, Ernst J. Rummeny, Dimitrios Karampinos, Rickmer F. Braren

**Affiliations:** 1 Institute for diagnostic and interventional Radiology, Klinikum rechts der Isar der Technischen Universität München, Ismaninger Straße, München, Germany; 2 Philips Healthcare, Hamburg, Germany; 3 Philips Healthcare, Best, Netherlands; McLean Hospital, UNITED STATES

## Abstract

**Objectives:**

To evaluate proton density fat fraction (PDFF) and T2* measurements of the liver with combined parallel imaging (sensitivity encoding, SENSE) and compressed sensing (CS) accelerated chemical shift encoding-based water-fat separation.

**Methods:**

Six-echo Dixon imaging was performed in the liver of 89 subjects. The first acquisition variant used acceleration based on SENSE with a total acceleration factor equal to 2.64 (acquisition labeled as SENSE). The second acquisition variant used acceleration based on a combination of CS with SENSE with a total acceleration factor equal to 4 (acquisition labeled as CS+SENSE). Acquisition times were compared between acquisitions and proton density fat fraction (PDFF) and T2*-values were measured and compared separately for each liver segment.

**Results:**

Total scan duration was 14.5 sec for the SENSE accelerated image acquisition and 9.3 sec for the CS+SENSE accelerated image acquisition. PDFF and T2* values did not differ significantly between the two acquisitions (*paired Mann-Whitney and paired t-test* P>0.05 in all cases). CS+SENSE accelerated acquisition showed reduced motion artifacts (1.1%) compared to SENSE acquisition (12.3%).

**Conclusion:**

CS+SENSE accelerates liver PDFF and T2*mapping while retaining the same quantitative values as an acquisition using only SENSE and reduces motion artifacts.

## Introduction

The non-invasive quantification of fat and iron content in liver tissue is of high clinical significance. For example, Non-Alcoholic Fatty Liver Disease (NAFLD) is the most common cause of chronic liver disease worldwide [1], with a prevalence of approximately 30% in adults in the western world [2]. In patients with NAFLD, liver damage results in hyperferritinemia and hepatic iron accumulation [3, 4]. Both hepatic iron overload and steatosis can result in fibrosis, progress to cirrhosis and therefore carry an increased risk for the development of hepatocellular carcinoma [5]. Despite availability of non-invasive imaging methods for quantification of hepatic fat and iron content, invasive tissue biopsy and histopathologic visualization of the fat deposition remains the gold standard in detection and quantification of hepatic steatosis and iron overload [6–12].

Magnetic resonance imaging (MRI) provides tools for fast, non-invasive quantitative imaging. Specifically, multi-echo gradient-echo acquisitions enable the simultaneous spatially-resolved mapping of the proton density fat fraction (PDFF) and T2*. Liver PDFF has emerged as a method for quantification of intrahepatic fat [13–17] with high sensitivity and specificity of 95.0% and 100.0% for the detection of histologic steatosis [13]. Due to its high diagnostic performance, PDFF is used as a reference modality for other methods of image-based fat quantification like computed tomography [18]. Likewise, liver T2* mapping has emerged as a method for quantification of intrahepatic iron content [19]. Chemical shift encoding-based water-fat separation by multi-echo gradient echo acquisition enables the simultaneous accurate and precise quantification of liver PDFF and T2*.

Parallel imaging has been traditionally used to reduce acquisition times, enabling chemical shift encoding-based water-fat separation measurements in a single breath-hold. However, further reduction of the breath-hold duration is highly desirable to avoid motion artefacts and thus improve accuracy of non-invasive fat and iron quantification, especially in patients with difficulty holding their breath. Compressed sensing (CS) allows for acceleration of MRI sequences and has been successfully utilized in various applications [20–22]. Some methodological works have employed CS for PDFF mapping and applied the technique in small volunteer or patient samples [23–26]. However, few studies exist on the performance of CS for simultaneous PDFF and T2* mapping in larger patient cohorts.

Therefore, the purpose of this study was the evaluation of the robustness of an CS-accelerated multi-echo gradient echo acquisition for the quantification of hepatic fat and iron content compared to a standard parallel-imaging-accelerated multi-echo gradient echo acquisition in a larger patient cohort.

## Material and methods

Approval by the institutional ethics committee (180/17S, Ethikkommission der Fakultät für Medizin der Technischen Universität München) was received for the study. The requirement to obtain written informed consent for retrospective data analysis was waived. All analyses were carried out in compliance with the pertinent regulations and requirements.

### Patient and public involvement

We did not involve patients or the public in our work.

### Patient cohort

We considered 217 datasets of patients who underwent routine clinical liver MRI examination from January 2018 until August 2018 for inclusion in the study. Datasets of patients with primary or secondary/metastatic liver tumors (N = 128) were excluded. The final patient cohort consisted of 89 patients (39 males and 50 females). Average patient age was 62.6±16.9 years (range 19–86 years). MRI was performed for the following indications: evaluation of focal pancreatic lesions (n = 57), pancreatitis (n = 15), evaluation of biliary lesions (n = 9) and suspected liver lesions (n = 8).

### Data acquisition

Two variants of multi-echo gradient-echo imaging for performing chemical shift encoding-based water-fat separation were performed sequentially on each patient at a 3 T MRI scanner (*Philips Ingenia Elition X; Philips Medical Systems*, Best, The Netherlands). The two acquisitions were based on a spoiled gradient echo sequence using bipolar gradient readouts. The first acquisition variant used acceleration based on SENSE with a total acceleration factor equal to 2.64 (acquisition labeled as SENSE). The second acquisition variant used acceleration based on a combination of CS with SENSE with a total acceleration factor equal to 4 (acquisition labeled as CS+SENSE). The relevant scan parameters are listed in *Table 1*.

**Table 1:** 

	Acquisition with SENSE	Acquisition with CS+SENSE
**Gating**	Breath-hold	Breath-hold
**Acquisition duration (s)**	14.5	9.3
**Acceleration factor**	2.2 x 1.2 = 2.64	4
**FOV (mm) FH; RL; AP**	150; 400; 300	150; 400; 300
**Acquisition voxel size (mm) FH; RL; AP**	6; 3; 2	6; 3; 2
**Reconstruction voxel size (mm) FH/RL/AP**	6; 1.14; 1.14	6; 1.14; 1.14
**Fast imaging mode**	none	none
**TE**_**eff**_ **/TE**_**equiv**_ **(ms)**		
**Act. TR (ms)**	7.8	7.8
**Act. TE (ms)**	1.35	1.35
**Flip angle (°)**	3	3

Scan parameters

The CS+SENSE technique used in the present work was based on the combination of SENSE and CS, labelled as Compressed SENSE or C-SENSE. The technique uses the coil sensitivity information from a SENSE calibration scan and randomly undersamples both the central and outer part of *k*-space, following a smooth sampling density as moving from the center to outer parts of k-space. The acquisition and reconstruction were based on the vendor’s implementation (Compressed SENSE, Philips Healthcare). A single CS acceleration factor was defined for the CS+SENSE acquisition variant and the sampled k-space pattern (central and outer part) was defined based on the vendor’s implementation. In order to maintain a balance between noise reduction and data consistency for CS, an iterative L1-minimization reconstruction technique, forcing data fidelity, and image sparsity in the wavelet domain, was used.

Complex multi-echo gradient-echo images were generated after the SENSE and CS+SENSE reconstructions and provided as input to the fat quantification routine provided by the vendor (*mDixon Quant*, Philips Healthcare). Specifically, after phase correction, a complex-based water-fat decomposition was performed using a single T_2_* correction and a pre-calibrated fat spectrum accounting for the presence of the multiple peaks in the fat spectrum. A seven-peak fat spectrum model was employed [27]. The PDFF map was computed as the ratio of the fat signal over the sum of fat and water signals.

### Image analysis

**Fig 1:**
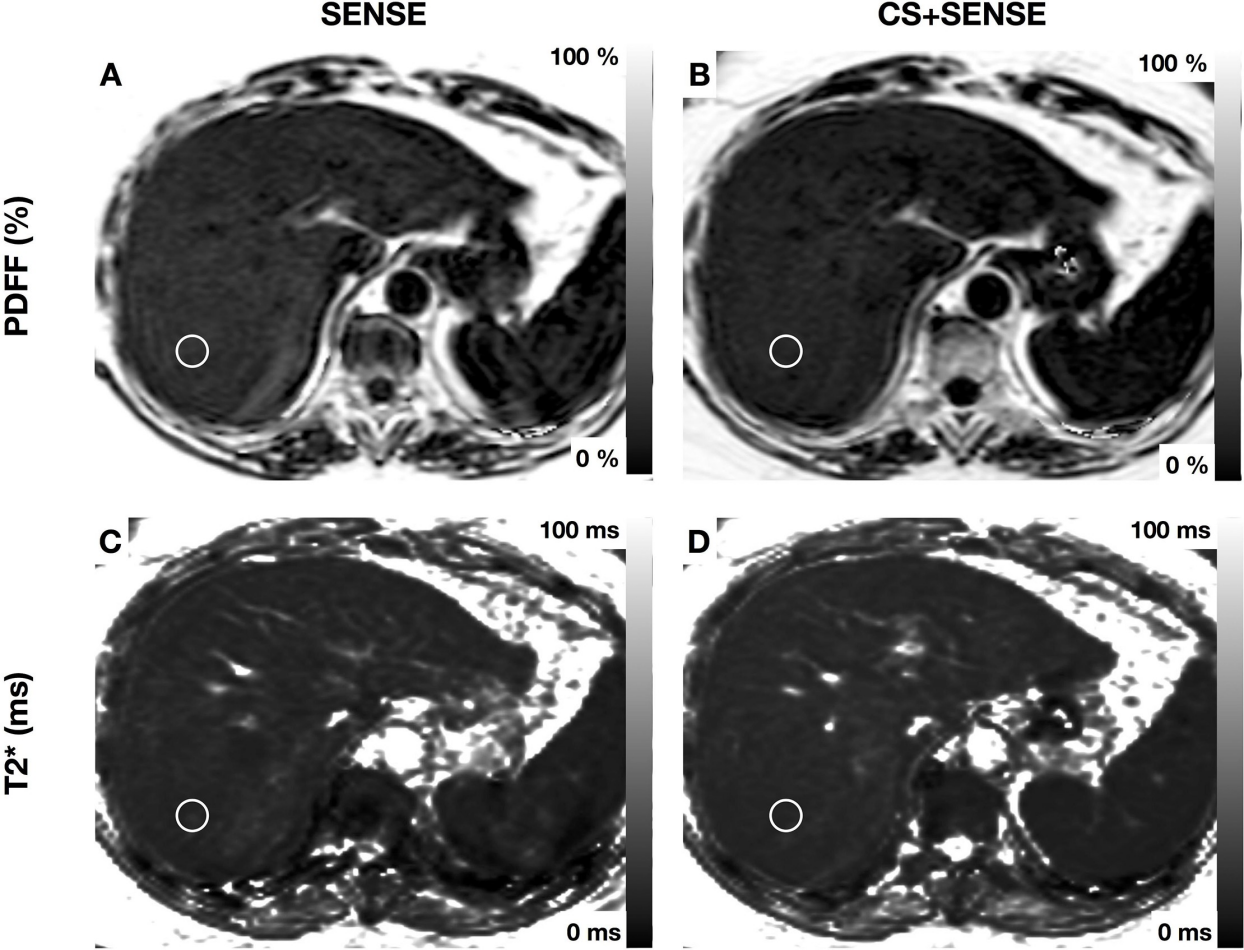
MRI was performed for evaluation of cystic pancreatic lesion. Exemplary region of interest is drawn in segment VII (1.5cm). PDFF: acquisition with SENSE 11.1±2.4% (A); acquisition with CS+SENSE 10.8±1.5% (B); T2*: acquisition with SENSE 9.35±0.6ms (C); acquisition with CS+SENSE 9.28±0.5ms (D).

Images were reviewed by two abdominal radiologists under standardized radiological reporting room conditions. Circular regions of interest (ROI) with a diameter of 15mm were manually drawn in each liver segment in consensus, avoiding large portal vein and hepatic vein branches (*Fig 1)*. T2* and PDFF maps were reviewed and mean ROI values and standard deviations were extracted. The software used was *Sectra IDS7 (*Linköping, Sweden). **Motion artifacts** were rated using a 4-point *Likert* scale as *1 = image not diagnostic because of artifacts; 2 = major artifacts; 3 = minor artifacts; 4 = no artifacts*.

### Statistical analysis

Variables were tested for normal distribution using the *D’Agostino-Pearson omnibus K2 test*. *Student’s t-test* was used for mean comparisons of normally distributed variables. The *Wilcoxon* test was used for mean comparisons of variables without normal distribution. All analyses were performed using *Prism* Version 7 (*GraphPad Software*). A two-tailed P-value below 0.05 was considered statistically significant.

## Results

The use of CS+SENSE accelerated image acquisition by 35% compared to the SENSE acquisition (from 14.5 to 9.3 seconds). Mean PDFF values ranged from 4.79±5.76% (liver segment IVb) to 6.37 ± 6.65% (liver segment VII) in the acquisition with SENSE and from 5.01±5.88% (liver segment IVb) to 6.28 ± 6.10% (liver segment VII) in the acquisition with CS+SENSE. Results did not differ significantly between the two acquisitions. All values are shown in *Table 2*. Mean PDFF was significantly higher in the right liver lobe compared to the left in both acquisitions (right lobe: 6.04 ± 6.36%; left lobe: 5.24 ± 5.69%; P = 0.03; acquisition with SENSE and right lobe: 5.96 ± 6.30%; left lobe: 5.16 ± 5.74%; P = 0.02; acquisition with CS+SENSE).

**Table 2:** 

Liver Segment	PDFF (%) in acquisition with SENSE	PDFF (%) in acquisition with CS+SENSE	p
**I**	5.36±4.83	5.11±4.49	0.1012
**II**	5.29±6.08	5.17±5.72	0.8659
**III**	5.04±5.68	5.01±5.88	0.9954
**IVa**	4.99±5.65	5.03±5.79	0.4.996
**IVb**	4.79±5.76	5.88±6.24	0.9889
**V**	5.53±6.72	5.50±6.73	0.5450
**VI**	6.19±5.93	6.05±5.76	0.1847
**VII**	6.37±6.19	6.28±6.10	0.7536
**VIII**	6.05±6.65	6.00±6.66	0.9425

PDFF mean values in % with standard deviation, no significant differences were seen between the two acquisitions. Wilcoxon test (no normal distribution); Patients: n = 89

**Fig 2:**
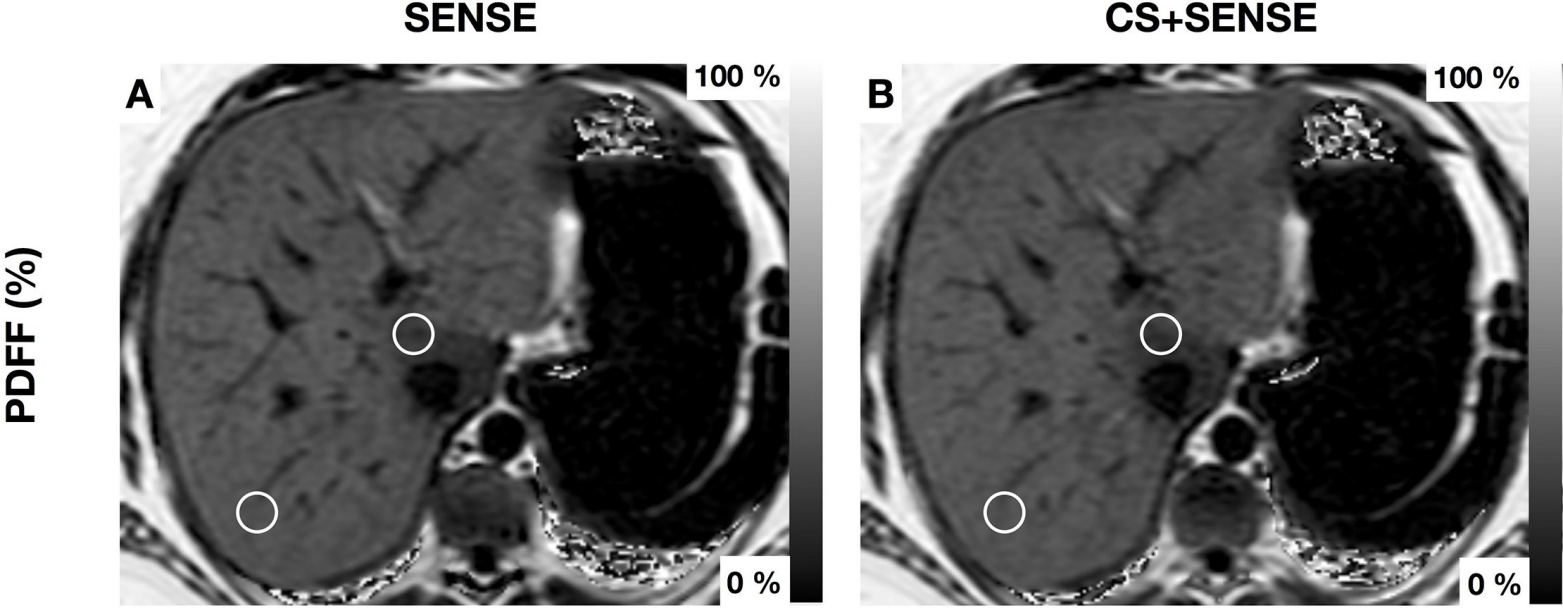
Patient with hepatic steatosis with focal fatty sparing of Segment I. Exemplary region of interest are drawn in Segments I and VII (1.5cm). PDFF Segment I: acquisition with SENSE 25.9±3.8% (A); acquisition with CS+SENSE CS4 25.8±4.5% (B) PDFF Segment VII: acquisition with SENSE 32.2±2.0% (A); acquisition with CS+SENSE 33.2±1.6% (B).

**Fig 3:**
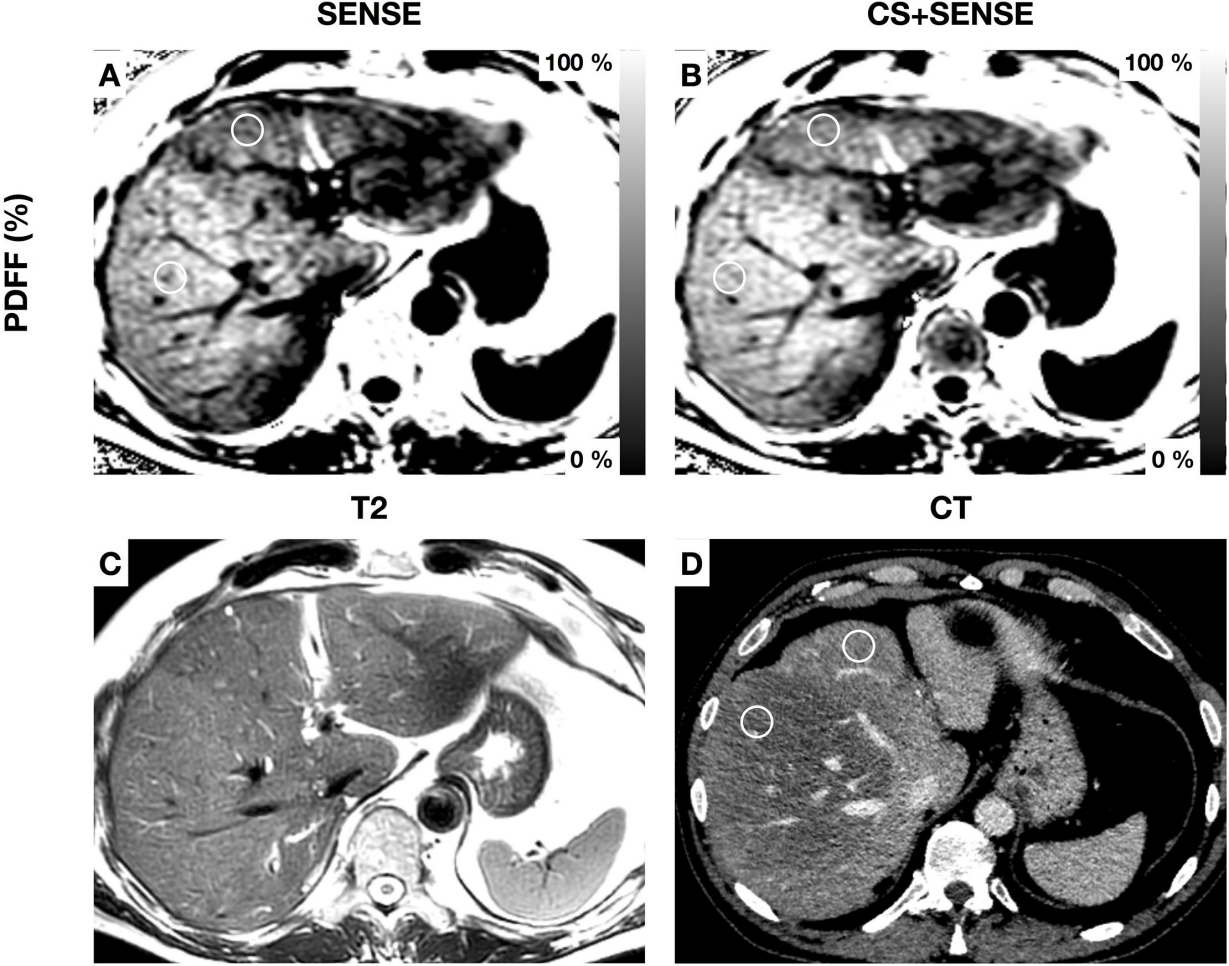
Patient with segmental hepatic steatosis. Exemplary regions of interest are drawn in segments IVa and V (diameter 1.5cm). PDFF Segment IVa: acquisition with SENSE 25.7±1.5% (A); acquisition with CS+SENSE 26.9±2.0% (B) PDFF Segment V: acquisition with SENSE 31.6±1.8% (A); acquisition with CS+ SENSE 32.6±2.2 (B) A T2-weighted image (C) and a CT image in the venous contrast phase (D) are shown for comparison. In CT Hounsfield units are 87±13 in Segment IVa and 42±16 in Segment V.

**Fig 4:**
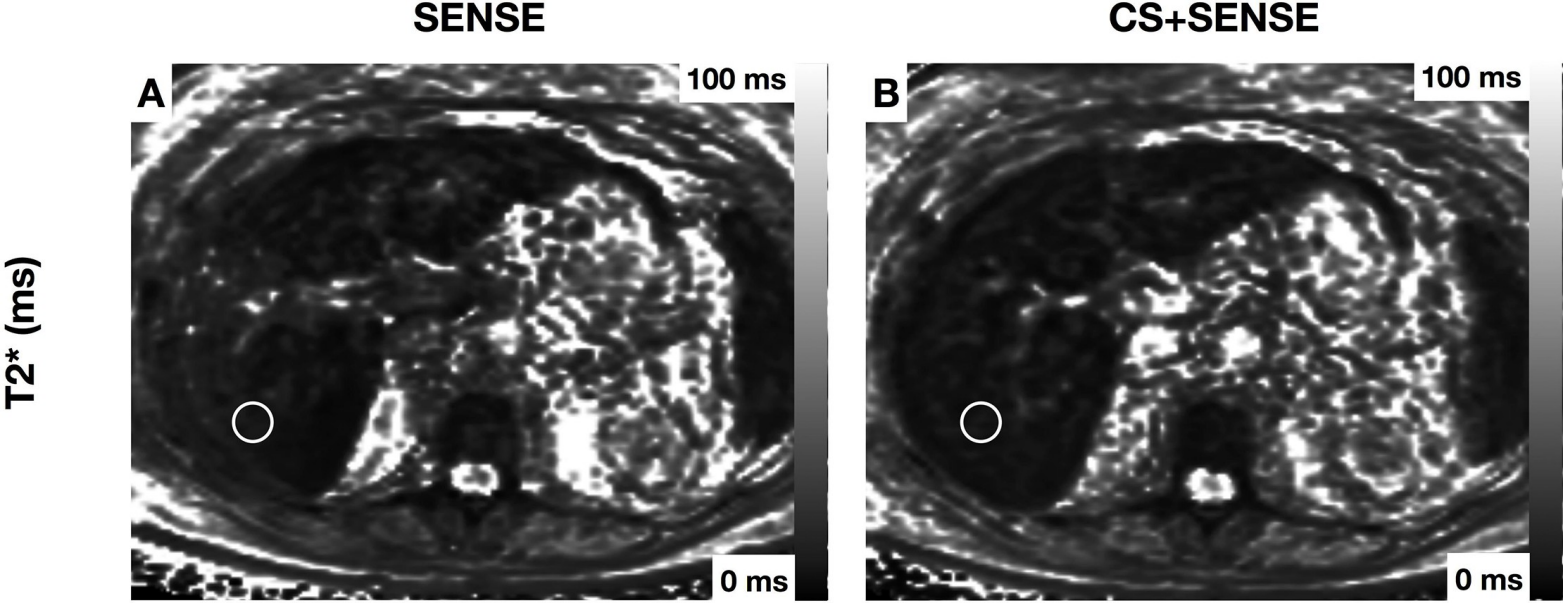
Patient with hemosiderosis and severe reduction of T2* Exemplary region of interest is drawn in segment VII (1.5cm). T2*: acquisition with SENSE 4.03±0.8ms (A); acquisition with CS+SENSE 5.56±1.0ms (B).

Mean T2* values ranged from 20.54±7.05 ms (liver segment II) to 23.42±7.43 ms (liver segment V) in the acquisition with SENSE and from 21.29±8.24 ms (liver segment II) to 23.30±8.60 ms (liver segment IVb) in the acquisition with CS+SENSE. T2* values did not differ significantly between the two acquisitions. T2* values with inter-patient standard deviation and results of the mean comparison are shown in *Table 3*. T2* values showed no significant difference between the right and left liver lobe in both acquisitions (right lobe: 22.60 ± 7.28ms; left lobe: 21.70 ± 7.14ms; P = 0.08 in the acquisition with SENSE; right lobe: 22.65 ± 7.31ms; left lobe: 22.14 ± 7.94ms; P = 0.35; in the acquisition with CS+SENSE). Images acquired with SENSE showed minor motion artifacts in 10.1% (n = 9) and major motion artifacts in 2.2% (n = 2) of the cases. Images acquired with CS+SENSE showed minor motion artifacts in 1.1% (n = 1) of all cases. One exemplary case with hepatic steatosis, sparing segment I is shown in *Fig 2. Fig 3* shows a patient with segmental steatosis. A T2 weighted image and a CT-image, acquired in the portal venous phase are shown as a comparison. *Fig 4* shows a case of a patient with hepatic iron overload due to hemosiderosis.

**Table 3:** 

Liver Segment	T2* (ms) in acquisition with SENSE	T2* (ms) in acquisition with CS+SENSE	p
**I**	22.25±7.70	23.04±7.67	0.1578
**II**	20.54±7.05	21.29±8.24	0.2096
**III**	20.96±6.98	21.38±7.85	0.3541
**IVa**	21.59±6.48	21.68±7.22	0.8428
**IVb**	23.15±7.30	23.30±8.60	0.7841
**V**	23.42±7.43	23.15±7.46	0.3549
**VI**	22.91±6.94	22.97±7.21	0.8399
**VII**	22.05±7.38	22.18±7.34	0.7391
**VIII**	22.05±6.76	22.32±7.31	0.4264

T2* mean values in ms with standard deviation no significant differences were seen between the two acquisitions. Paired t test (normal distribution); Patients: n = 89

## Discussion

The data presented in this study show comparability of quantitative PDFF and T2* measurements acquired with compressed sensing (CS)-accelerated chemical shift encoding-based water-fat separation. Our results demonstrate a significantly higher PDFF in the right lobe of the liver, which is in accordance with other studies [28, 29], and no spatial dependence of liver T2* values.

The most important finding of the presented data is the agreement of the quantitative PDFF and T2* imaging results between the two acquisitions. Several studies have shown high accuracy of MRI-based imaging techniques for the non-invasive quantification of fat and iron content of the liver [13, 14, 29]. Due to their widespread availability, chemical shift encoding-based water-fat separation techniques can be used as a fast screening for NAFLD or disorders of iron metabolism. Given the increase of medical imaging in the last years [30], the acquisition time is an essential factor. In our study, CS+SENSE was able to accelerate image acquisition of liver PDFF and T2* mapping by 35% to a scan time of only 9.3 seconds. In a liver segment-based comparison between the acquisition with CS+SENSE and the acquisition with SENSE, no significant differences were detected in the quantitative parameters PDFF and T2*. Thus, reduction of scan time lead to a reduction of motion artifacts and did not lead to changes in quantitative parameters, rendering the presented chemical shift encoding-based water-fat separation technique combined with CS+SENSE a sequence with an excellent applicability in routine MRI liver examinations. The widespread availability could allow an application for large studies on hepatic steatosis.

Previous methodological works applied CS for the joint problem of image reconstruction and water-fat separation [23–25]. The present work estimates the PDFF and T2* maps in two steps: it first applies the CS+SENSE reconstruction for the reconstruction of the multi-echo complex images and then applies water-fat separation on the reconstructed water-fat images, as also previously performed [26, 31]. Although higher acceleration factors can be achieved by solving the joint step instead of solving the problem in two steps, the present study shows that a prospective CS+SENSE accelerated acquisition using the two-step approach already results in reliable PDFF and T2* maps in the presently studied patient cohort.

Our study has some limitations. First, the study was only performed in one center and on one scanner to show the feasibility of the accelerated acquisition with CS+SENSE. We did not test the two acquisitions at different field strengths. Second, histologic correlation was not performed. However, this was not within the scope of the current study that aimed at showing robustness of quantitative parameters when using CS acceleration. Former studies have have already shown an accurate estimation of liver fat by PDFF in correlation with histologic findings [32, 33]. In addition, several previous studies have already shown high sensitivity and specificity in liver quantitative imaging with chemical shift encoding based water-fat separation techniques accounting for the same confounding factors [13, 34].

In conclusion, the acceleration of chemical shift encoding-based water-fat separation using compressed sensing results in comparable quantitative measurements of hepatic fat and iron content with reduced breath-hold intervals, leading to reduced motion artifacts and making chemical shift encoding-based water-fat separation a fast and precise non-invasive tool in quantitative liver imaging.

## Supporting information

S1 Table (XLSX)
